# FAITH IN ACTION BRAIN HEALTH PROGRAM: ADDRESSING BRAIN HEALTH EQUITY GAPS IN THE BLACK COMMUNITY

**DOI:** 10.1093/geroni/igad104.0569

**Published:** 2023-12-21

**Authors:** Fayron Epps, Mia Chester, Janelle Gore, Glenna Brewster, Mayra Sainz, Robbin Frazier, Stephanie Monroe, Virginia Biggar

**Affiliations:** Emory University, Atlanta, Georgia, United States; Emory University, Atlanta, Georgia, United States; Centers for Disease Control and Prevention, Atlanta, Georgia, United States; Emory University, Atlanta, Georgia, United States; Emory University, Atlanta, Georgia, United States; University of Minnesota, Center for Healthy Aging and Innovation, Minneapolis, Minnesota, United States; UsAgainstAlzheimer’s, Washington, District of Columbia, United States; UsAgainstAlzheimer’s, Washington, District of Columbia, United States

## Abstract

Black populations are disproportionately impacted by dementia, being twice as likely to be diagnosed with dementia as White people. To help address brain health equity gaps across the country, a partnership between Alter and UsAgainstAlzheimer’s BrainGuide was established to launch, Faith in Action, a campaign and brain health education program to provide culturally appropriate brain health resources to Black faith communities. The campaign makes brain health education free and accessible by integrating the latest information about brain health into a toolkit delivered to faith communities. This presentation will describe the campaign and discuss why faith leaders say brain health is important to Black faith communities. This campaign included the distribution of: a) Fact sheets b) Culturally tailored messaging; c) Church fans, bookmarks, and buttons; and d) A guidebook to integrating brain health messaging. In relation to the importance of brain health, the following 5 themes emerged from faith leaders (N = 33) implementing the Alter program: a) Desire to disrupt statistics; b) Urgency and being proactive; c) Offer valuable intergenerational education programs; d) Establish a level of wellness in underserved communities; and e) Protect older adults. The Faith in Action campaign empowers Black faith communities to foster conversations and educate their community members about brain health. This campaign assists in promoting brain health equity by recognizing the significance of partnering with Black churches, a cornerstone of the Black community. Such partnerships are crucial to increasing equitable access to resources and supportive services, and eradicating dementia-related disparities in the Black community.

